# Assessment of GO-Based Protein Interaction Affinities in the Large-Scale Human–Coronavirus Family Interactome

**DOI:** 10.3390/vaccines11030549

**Published:** 2023-02-25

**Authors:** Soumyendu Sekhar Bandyopadhyay, Anup Kumar Halder, Sovan Saha, Piyali Chatterjee, Mita Nasipuri, Subhadip Basu

**Affiliations:** 1 Department of Computer Science and Engineering, Jadavpur University, Kolkata 700032, India; 2 Department of Computer Science and Engineering, School of Engineering and Technology, Adamas University, Kolkata 700126, India; 3 Faculty of Mathematics and Information Sciences, Warsaw University of Technology, 00-662 Warsaw, Poland; 4 Department of Computer Science and Engineering (Artificial Intelligence and Machine Learning), Techno Main Salt Lake, Sector V, Kolkata 700091, India; 5 Department of Computer Science and Engineering, Netaji Subhash Engineering College, Kolkata 700152, India

**Keywords:** COVID-19, SARS-CoV-2, COVID-19 variants, go-semantic score, gene ontology, COVID-19 drugs, protein–protein interaction network

## Abstract

SARS-CoV-2 is a novel coronavirus that replicates itself via interacting with the host proteins. As a result, identifying virus and host protein-protein interactions could help researchers better understand the virus disease transmission behavior and identify possible COVID-19 drugs. The International Committee on Virus Taxonomy has determined that nCoV is genetically 89% compared to the SARS-CoV epidemic in 2003. This paper focuses on assessing the host–pathogen protein interaction affinity of the coronavirus family, having 44 different variants. In light of these considerations, a GO-semantic scoring function is provided based on Gene Ontology (GO) graphs for determining the binding affinity of any two proteins at the organism level. Based on the availability of the GO annotation of the proteins, 11 viral variants, *viz.*, SARS-CoV-2, SARS, MERS, *Bat coronavirus* HKU3, *Bat coronavirus* Rp3/2004, *Bat coronavirus* HKU5, *Murine coronavirus*, *Bovine coronavirus*, Rat coronavirus, *Bat coronavirus* HKU4, *Bat coronavirus* 133/2005, are considered from 44 viral variants. The fuzzy scoring function of the entire host–pathogen network has been processed with ~180 million potential interactions generated from 19,281 host proteins and around 242 viral proteins. ~4.5 million potential level one host–pathogen interactions are computed based on the estimated interaction affinity threshold. The resulting host–pathogen interactome is also validated with *state-of-the-art* experimental networks. The study has also been extended further toward the drug-repurposing study by analyzing the FDA-listed COVID drugs.

## 1. Introduction

The emerging coronavirus (CoV) pandemic has sparked a flurry of research into the SARS-CoV-2 virus and the COVID-19 disease it causes in people [1]. COVID-19 was identified in Wuhan (Hubei province) [2]. It starts spreading soon to other nations. On 30 January 2020, World Health Organization (WHO) declared this outbreak of nCoV as a global emergency [3]. A coronavirus is a member of the family Coronaviridae.

Along with humans, it also affects mammals and birds. Even though the coronavirus typically causes the common cold, cough, etc., it also causes severe acute, chronic respiratory disease, multiple organ failure, and, ultimately, human mortality. Before SARS-CoV-2, the two primary outbreaks were Middle East Respiratory Syndrome (MERS) and Severe Acute Respiratory Syndrome (SARS). Southern China was the location of SARS’s inception. Its fatality rate was between 14 and 15% [4]. The MERS outbreak was supposed to start in Saudi Arabia. In the fight against the MERS virus, 858 out of 2494 afflicted cases prevailed. As a result, it produced a substantially higher death rate of 34.4% compared to the SARS.

Regarding biology, the three epidemic-starting viruses, SARS, MERS, and SARS-CoV-2, belong to Coronaviridae’s genus Beta coronavirus. Proteins that are both structural and non-structural contribute to the development of SARS-CoV-2. Out of the two, structural proteins such as the spike (S) protein, nucleocapsid (N) protein, membrane (M) protein, and envelope (E) protein play a crucial part in spreading the disease by binding with receptors after entering the human body [5]. 

The primary factor which needs to be considered while examining the disease transmission process from SARS-CoV-2 to humans is the Protein–Protein Interaction Network (PPIN). It is critical for determining essential proteins and functions [6,7,8,9,10,11,12,13,14,15,16,17,18,19] responsible for various diseases. The primary focus of research has changed from the study of the PPIN underlying various types of human diseases to the study of the PPIN due to the improvement in the availability of human PPIN data [20]. According to the report, SARS-CoV-2 has ~89% similarity with SARS-CoV [21,22]. SARS-CoV, a disease that initially appeared in the Guangdong Province of China in November 2002, spread to 28 regions worldwide in 2003 and resulted in 774 fatalities among the 8096 people with COVID-19 [23,24,25]. According to phylogenetic analysis, it was assumed that SARS-CoV was different from previously known coronaviruses [26,27]. Even though the etiological agent was discovered and molecular research on the SARS-CoV advanced quite quickly, the mystery surrounding the disease’s cause remained unsolved. Data indicated that SARS was an animal-borne disease from the beginning [23,24,28,29]. After the surge of SARS-CoV in 2012, there was another coronavirus surge, Middle East Respiratory Syndrome (MERS), in Jordon. A bat and numerous dromedary camels have been reported to have MERS-CoV sequences (DC). MERS-CoV is an enzootic disease in the Arabian Peninsula, portions of Africa, and the Middle East. It affects camels as its primary reservoir and occasionally, but infrequently, infects humans [30]. MERS-CoV is a member of the Beta coronavirus family. World Health Organization (WHO) confirmed 2220 people with COVID-19 along with 790 deaths for MERS-CoV [31]. There is a 35% fatality rate from MERS. MERS is not specifically treated. MERS-CoV outbreaks in hospitals and homes are brought on by person-to-person transmission [32]. 

A beta-CoV prevalent in wild mice, the mouse hepatitis virus (MHV) or Murine-CoV is similar to SARS-CoV-2. In-depth research has been done on laboratory MHV strains to understand host antiviral defense systems and coronavirus virulence factors [33]. Murine-CoV contains several strains that induce variable symptoms in the respiratory, digestive, hepatic, and neurological systems [34,35,36]. The genus of beta-CoVs includes all MHV strains and certain human CoVs (HCoV-OC43, HCoV-HKU1, SARS-CoV, MERS-CoV, and SARS-CoV-2). The tropism and pathogenicity of various MHV strains vary, and research on recombinant MHV variations has uncovered host and viral variables that affect viral propagation or evade immune Identification [37].

The wide variety of mammalian and avian species that coronaviruses have been found to infect and the highly varied disease syndromes they cause are well known. One of the well-known traits of several CoVs is variable tissue tropism, which also allows them to overcome interspecies boundaries easily. Betacoronaviruses, known as bovine CoVs (BCoVs), cause shipping fever, winter dysentery in older cattle, and neonatal calf diarrhea. Interestingly, there have not been any specific genetic or antigenic markers found in BCoVs linked to these unique clinical disorders. BCoVs, on the other hand, are quasispecies that coexists with other CoVs. In addition to cattle, BCoVs and CoVs resembling cattle were found in several domestic and wild ruminant species, dogs, and humans [38]. The pneumoenteric virus known as the bovine coronavirus (BCoV) is a member of the Betacoronavirus 1 genus. Because of several instances of genetic recombination and interspecies transmission, members of the Betacoronavirus 1 species appear to be host-range variants descended from the same parental virus due to their close antigenic and genetic relatedness [39,40,41,42].

Two separate teams reported finding SARS-like CoVs (SL-CoVs) in bats in 2005, and they hypothesized that bats were SARS-CoV natural reservoirs [43,44]. Most bat SL-CoVs were discovered in rhinolopus bats, especially Rhinolophus sinicus. They share 87 to 92% of their nucleic acid and 93 to 100% of their amino acid sequences with the SARS-CoV [43,44,45,46,47]. According to a phylogenetic study, MERS-CoV is a member of lineage C of the Betacoronavirus genus. It resembled the pipistrelle bat (Pipistrellus pipistrellus) and lesser bamboo bat (*Tylonycteris pachypus*) most closely, as well as the bat coronaviruses HKU4 and HKU5 [31,48]. The whole genomic sequences of HKU4 and HKU5 and the RNA-dependent RNA polymerase (RdRp) gene show nucleotide identity with MERS-CoV of 50% and 82%, respectively. A recent study established that CD26, also known as dipeptidyl peptidase 4 (DPPIV), is a functional receptor for MERS-CoV. Additionally, it has been demonstrated that this molecule is evolutionarily conserved among mammals and that MERS-CoV can infect a wide variety of mammalian cells (including those from humans, pigs, monkeys, and bats), indicating ease of transmission between hosts [49,50].

A large-scale PPI network of an organism provides valuable clues for understanding cellular and molecular functionalities, and signaling pathways can provide crucial insights into the disease mechanism, etc. Much biological information is available and encoded in different ontologies called Gene Ontology. Semantic similarity is the degree of relatedness between the two biological entities (Gene/Protein) based on GO annotations that provide a quantitative measure of their GO-level relationship [51]. Different combinations of edge-based and node-based semantic similarity measures have been applied over the years from gene ontology graphs [52,53,54,55,56,57,58,59,60,61,62,63]. These methods have specific shortcomings concerning their designed GO semantic features. Some of them have used topological properties of the GO graph, some have used only the information content (IC) of the most informative common ancestor [52,53,55,56], and some have used DCA [58,59,60] based approach. To define the interaction affinity of any two proteins from their GO information, this hybrid approach is more effective as it incorporates topological features and average IC-based DCA techniques. Much work [64] has already been done to analyze host–pathogenic interactions [65,66], disease detection [67], and disease-specific multi-omics network analyses [68].

From the above discussion, it is clear that several similar studies based on GO information have been done on host–pathogen interaction networks. However, a complete PPIN must be identified for humans and different coronavirus organisms to detect probable human targets from all perspectives. So, in this study, the interaction affinity between the protein pairs from the different organisms of the coronavirus family and human spreader proteins is calculated using the available ontological information using the proposed in-silico model. Section 2 describes the proposed in-silico model for calculating the interaction affinity of the bait-prey protein pairs in an apache spark-based parallel computational environment. Section 2.2 gives a detailed description of the database used for different coronavirus organisms. The results are discussed in Section 3, which includes host–pathogen protein interactions for the different organisms of the coronavirus family and validation of our proposed in-silico model using the *state-of-the-art* database.

## 2. Materials and Methods

A GO-based Graph theoretic model is proposed to determine the interaction affinity between the host–pathogen protein pairs for humans and different coronavirus organisms. Currently, 19,281 human proteins have GO annotations, whereas around 242 viral proteins are obtained from a selected organism having GO annotations. Based on the above data, level 1 interactors generates ~4.5 million potential host–pathogen interaction. The variety and veracity issue plays a significant role in such a large-scale dynamic PPI network. Handling large, dynamic, heterogeneous networks using in-silico methods is tedious. Therefore, an Apache Spark-Based analytical study is proposed to compute the interaction affinity in large-scale protein–protein interaction networks using the Gene Ontology (GO) graph.

### 2.1. GO Graph-Based Scoring for Potential Host–Pathogen Protein Interaction Identification

Combining the similarity scores of the GO terms connected to the proteins will yield an estimate of the semantic similarity between two interacting proteins [52,66,69,70]. The greater the similarity between two GO pairs, the greater the interaction affinity between the proteins. The GO hierarchy’s independent directed acyclic graphs (DAGs) represent three distinct features of proteins: cellular component (CC), biological process (BP), and molecular function (CC). Each node represents GO terms, and edges indicate various hierarchical relationships. The two fundamental relations *“is_a”* and *“part of”* GO graphs are considered for semantic score computation. Considering the similarity between all the GO pairs, the semantic similarity of the protein pairs can be estimated. The shortest path length between a pair of terms in a GO graph and the average information content *(IC)* [57] of the disjunctive common ancestors (*DsjCA*) of the respective GO term [52,70] measures the similarity of the pair. Our proposed method based on the GO graph is fuzzy clustered, and the degree of relationship between each GO term and the cluster center determines which GO term is chosen as the cluster center. The cluster centers are then chosen using the GO term proportion measure. The proportion measure of any GO term t is given by

(1) $$\mathrm{PrT}\left(t\right)=\frac{\left|AnC\left(t\right)\right|+\left|DnC\left(t\right)\right|}{\left|No\right|}$$

where *AnC(t)* is the ascendant term for t and *DnC(t)* is the descendent term of t. *No* is the total number of GO terms in ontology O, and *PrT(t)* is the proportion measure of term t. The GO keywords chosen as cluster centers are those for which this proportion metric is higher than a certain threshold. The cluster centers in this study are selected using the proposed threshold values [66,69]. Once the cluster centers have been chosen, the shortest path lengths between each term in the ontology and the cluster centers have been calculated. The membership value of a GO term decreases with the increase in the shortest path length. The membership function of a GO term is given by

(2) $$Mfn_{c}\left(t\right)=e^{-\frac{-{\left(x-c_{i}\right)}^{2}}{2k^{2}}}$$

where *c_i_* is the *i^th^* cluster center, *x* is the shortest path length, and *k* is the width of the membership function. If no path from any GO term to a cluster center is found, then the membership of the GO term with respect to that cluster center will be considered 0. Similar membership for any target GO pair indicates very closely related concepts of GO functionality, and widely related membership value represents separated concepts. For any target pair of GO term (*t_i_*,*t_j_*), a weight parameter is introduced to estimate these differences in membership. The weight parameter is thus defined by

*WT(t_i,_ t_j_) = 1 − maxD (t_i_,t_j_)*

where *maxD*(*t_i_*,*t_j_*) represents the maximum difference in membership values of GO pair (*t_i_*,*t_j_*) across all cluster centers of any particular GO graph type(CC/MF/BP).

The information content (*IC*) based information of the disjunctive common ancestor (*DsjCAs*) of any GO graph is more significant in the semantic similarity assessment of two GO terms [60]. *IC* of any GO term t, with respect to a GO graph, g is defined as *ICg(t) = −log(Pr(t))*. The probability *Pr(t)* is the occurrences of term t with respect to the total annotations of GO graph g. The occurrences of term *t* depend on its annotations over the protein corpus. Using the *IC* of the *DsjCA*, the shared information content (*SIC*) is computed for the target GO term pair (*t_i_*,*t_j_*). The SIC is computed as

(3) $$SIC\left(t_{i},t_{j}\right)=\frac{\Sigma a\in DsjCA^{IC\left(a\right)}}{\left|DsjCA\left(t_{i},t_{j}\right)\right|}$$

Finally, the semantic similarity between two GO pair *t_i_* and *t_j_* is calculated as

(4) $$\mathrm{SS}t_{i}t_{j}=\mathrm{WT}(t_{i},t_{j})\times \;SIC\left(t_{i},t_{j}\right)$$

**Figure 1:**
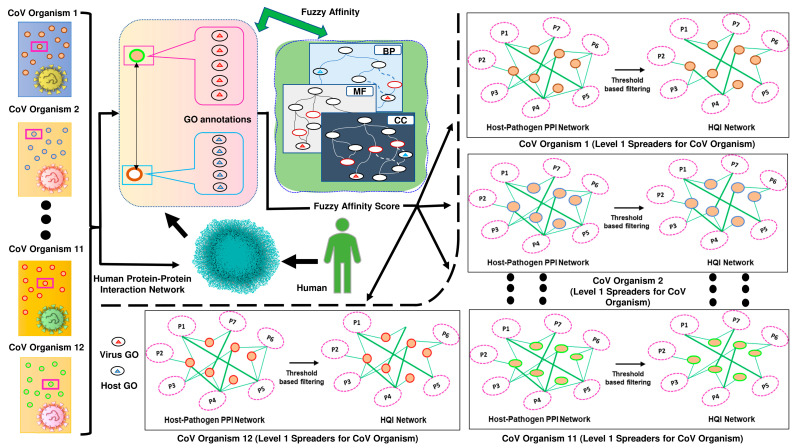
Schematic diagram of our proposed model. The coronavirus and human proteins’ interaction affinities are determined by the model using gene ontology information of the proteins. Three different GO-relationship graphs, CC, MF, and BP, are used to evaluate all GO pair-wise interaction affinities. A protein pair’s fuzzy interaction affinity is calculated using the three pair-wise scores of all GO-pair affinities.

When comparing the annotations of the proteins *P_i_* and *P_j_* for each type of GO, the maximum similarity of all possible GO pairs is used to determine the semantic similarity of the protein pair (*P_i_*, *P_j_*) for each GO type (CC, MF, and BP). The average of the CC, MF, and BP-based semantic similarity is used to define the protein pair’s interaction affinity (*P_i_*, *P_j_*). Figure 1 refers to the schematic diagram of our proposed model where the host–pathogen interaction affinity between humans and organisms from the coronavirus family is calculated using the GO information, resulting in high-quality interactions for retrieving vulnerable human prey for coronavirus hosts.

### 2.2. Dataset Preparation

Alpha-, Beta-, Gamma-, and Delta-CoV are the four genera that comprise the enormous family of enveloped positive-strand RNA viruses known as coronaviruses (CoVs). Among all the 44 organisms of coronavirus, here in this work, only 11 organisms have been considered based on the available GO-annotated proteins. The human is considered the host, and the work mainly suggests the affinity of host–pathogen interaction for different coronavirus organisms. Below, a brief description of all selected organisms is given.

#### 2.2.1. Human Protein

All potential interactions between human proteins that have been experimentally verified in humans make up the dataset [71,72]. The proteins in the Human organism are represented by nodes, whereas the edges represent the respective interactions between the organism. The proteins and their GO annotations are collected from UniProt, the protein repository [73]. UniProt contains 20,386 reviewed human proteins, among which 19,283 proteins are associated with GO annotations. 

#### 2.2.2. SARS-CoV-2 Proteins

SARS-CoV-2 is a biological member of the Coronaviridae, which belongs to the genus Beta coronavirus. The virus contains four structural proteins, namely envelop(E) protein, membrane(M) protein, nucleocapsid(N) protein, and spike(S) protein, which helps in binding with receptors after entering the human body and has a crucial function in spreading the disease [5]. Here the work is carried out by collecting the dataset of available SARS-CoV-2 protein from UniProtKB. The repository includes 16 reviewed SARS-CoV-2 proteins as of date.

#### 2.2.3. SARS-CoV Proteins

SARS-CoV is a highly pathogenic and zoonotic virus that causes severe respiratory illness, gastrointestinal, neurological, and fatalities among humans [74,75,76]. The 2002-2003 severe acute respiratory syndrome (SARS) pandemic showed how susceptible humans are to CoV epidemics [77]. However, the dataset is collected from UniProtKB, which holds 15 reviewed SARS-CoV proteins.

#### 2.2.4. MERS-CoV Proteins

MERS-CoV is also a member of Beta-Coronavirus. It is an even more pathogenic and zoonotic virus in comparison to SARS-CoV. MERS-CoV immerged around 2012 in the Arabian Peninsula with very high transmissibility by affecting more than 2000 people [78]. The dataset has been retrieved from UniProtKB, which holds around 10 MERS-CoV proteins.

#### 2.2.5. *Bat coronavirus* HKU3 Proteins

Surveillance research in Hong Kong among non-caged animals from wild regions found that a closely similar bat coronavirus, SARS-related Rhinolophus bat coronavirus HKU3, was the natural animal host [79]. We have retrieved a protein set of *Bat coronavirus* HKU3 from UniProtKB, having 12 proteins.

#### 2.2.6. *Bat coronavirus* RP3/2004 Proteins

With the high geographic spread and species variety, bats represent an order with significant evolutionary success. Bats are the natural reservoirs of several viruses closely related to SARS-CoV [80]. A search for ACE2 sequence similarities in domestic and wild animals in Italy revealed domestic (horses, cats, cattle, and sheep) and wild (European rabbits and grizzly bears) animal species as potential SARS-CoV-2 secondary reservoirs. Molecular docking of these species’ ACE2 against the S protein of the *Bat coronavirus* (Bt-CoV/Rp3/2004) suggests that the primary reservoir *Rhinolophus ferrumequinum* may infect secondary reservoirs, domestic and animals living in Italy [81].

#### 2.2.7. *Bat coronavirus* HKU5 Proteins

An enclosed, positive-sense single-stranded RNA mammalian Group 2 Betacoronavirus called bat coronavirus HKU5 (Bat-CoV HKU5) was found in Japanese Pipistrellus in Hong Kong. This coronavirus strain is closely related to the recently discovered novel MERS-CoV, which is to blame for the coronavirus outbreaks linked to the Middle East respiratory illness in 2012 [31,82]. 

#### 2.2.8. *Bat coronavirus* HKU4 Proteins 

Tylonycteris bat coronavirus HKU4 (Bat-CoV HKU4), a member of Betacoronavirus, is an enveloped, single-stranded virus having a genetical similarity with MERS-CoV or HCoV-EMC. The main difference between HCoV-EMC and Bat-CoV HKU4 lies in between the spike protein (S) and envelop (E) protein, where HCoV-EMC have five ORFs instead of four with low amino acid identities to Bat-CoV HKU4 [83]. The human CD26 (hCD26) receptor is engaged explicitly by a receptor binding domain (RBD) in the MERS-CoV envelope-embedded spike protein to start viral entry. Due to the viral spike protein’s great sequence identity, we looked into whether or not HKU4 and HKU5 can detect hCD26 for cell entrance. We discovered that HKU4-RBD binds to hCD26, but not HKU5-RBD, and that pseudotyped viruses incorporating HKU4 spike can infect cells by recognizing hCD26. The overall hCD26-binding mechanism of the HKU4-RBD/hCD26 complex was identical to that of the MERS-RBD, according to the structure. However, HKU4-RBD has a lower affinity for receptor binding than MERS-RBD because it is less suited to hCD26 [84]. 

#### 2.2.9. *Bat coronavirus* 133/2005

The spike (S1) and RNA-dependent RNA polymerase proteins of MERS-CoV were subjected to phylogenetic analysis, which indicated that the virus is linked to bat viruses. Coronavirus surveillance investigations in several populations of bats have shown that they are potential reservoirs for this unique virus [85]. Different phylogenetic studies reveal that MERS-CoV was grouped with the Betacoronavirus genus, particularly near BtCoV/133/2005 and BtCoV HKU4-2, which had the most significant S1 amino acid sequence similarity (60%) with MERS-CoV [86].

#### 2.2.10. *Murine coronavirus*

*Murine coronavirus* (M-CoV), a member of the Betacoronavirus family having Embacovirus subgenus, is mainly found responsible for infecting rats [87,88]. Enterotropic and Polytropic are the two strains of M-CoV. Mouse hepatitis virus (MHV) strains D, Y, RI, and DVIM are examples of enterotropic strains. In contrast, hepatitis, enteritis, and encephalitis are the leading causes of illness caused by polytropic strains like JHM and A59 [89]. *Murine coronaviruses* come in over 25 distinct strains. These viruses, which spread by the fecal-oral or respiratory routes and infect mice’s livers, have been utilized as an animal disease model for hepatitis [90]. The strains MHV-D, MHV-DVIM, MHV-Y, and MHV-RI, which are transmitted in fecal matter, primarily affect the digestive tract. However, they can occasionally affect the spleen, liver, and lymphatic tissue [91]. 

#### 2.2.11. *Bovine coronavirus*

*Bovine coronavirus* (BCoV) is a member of Betacoronavirus 1, and it can infect both cattle and humans [92,93]. It is also an enveloped single-stranded RNA virus that enters the host cell by binding itself with the N-acetyl-9-O-acetylneuraminic acid receptor [94,95]. BCov is mainly responsible for causing gastroenteritis in calves resulting in massive economic damage [96]. BCoV consisted of five structural proteins, namely (S) spike glycoprotein; (M) integral membrane protein; (HE) hemagglutinin-esterase glycoprotein; (E) small membrane protein, and (N) nucleocapsid phosphoprotein [97]. A phosphoprotein with a high content of essential amino acids, the N protein joins the genomic RNA directly to create a helicoidal nucleocapsid. The N protein carries out numerous activities related to viral pathogenicity, transcription, and replication. Because it is a highly conserved protein expressed in significant amounts during viral replication, it is frequently employed for molecular diagnosis of BCoV [98].

#### 2.2.12. *Rat coronavirus*

*Rat coronavirus* (RCoV), subset of *Murine coronavirus*, is also a single stranded RNA virus belonging to Betacoronavirus family which is responsioble for infecting rats [99]. The respiratory disease in adult rats is caused by RCoV in adult rats, which is characterized by an early Polymorphonuclear neutrophils (PMN) response, viral multiplication, inflammatory lung lesions, modest weight loss, and efficient infection resolution [100]. When a virus is present, PMN in the respiratory tract is typically associated with severe disease pathology [101,102,103,104].

## 3. Results

Our developed in-silico model contains the protein interaction affinity between humans and different organisms from the coronavirus family. The in-silico model is validated by identifying the overlapped edges with reference to the *state-of-the-art* datasets. Any computational model must always consider the input and output source, and our suggested model is no exception.

### 3.1. Identification of Host–Pathogen Protein Interactions for the Different Organisms of the Coronavirus Family

Three different forms of GO hierarchical connection graphs can be used to use the GO information to infer the binding affinity of each pair of interacting proteins (CC, MF, and BP) [64]. Our proposed GO-based in-silico model is applied to find the interaction affinity between the host protein and different organisms of the coronavirus family. Among 44 different organisms of the coronavirus family, based on the availability of the proteins, 11 organisms are considered. Our model is created from the ontological relationship graphs by comparing the affinities of all potential GO pairings that may be annotated from any target protein pair. Finally, the score of interaction affinity of protein pair based on their annotated GO pair-wise interaction is computed within a range of [0, 1]. Table 1 gives a detailed description of the number of proteins available for the respective coronavirus organism and the number of possible host–pathogen interaction networks that can be generated for each organism.

### 3.2. Detailed Description of Human–nCoV Protein Interaction Network

The 2019 coronavirus disease pandemic was brought on by the novel coronavirus known as severe acute respiratory syndrome coronavirus 2 (SARS-CoV-2/nCoV). It affected over 12 million people and caused over 560,000 fatalities in 213 nations [105]. To infect a host, the nCoV protein, like other virus proteins, must interact with the host protein and replicate the genome. Detailed descriptions for all types of possible interactions are given in Table 2. At the time of our experiment, UniProt [106] holds around 19,283 human proteins and 16 nCoV proteins (Table 3) having GO annotations. Here, through our proposed in-silico model, we compute all the possible protein interactions between human-nCoV for all the proteins having GO annotations (Table 4). Here ‘Total Dataset’ refers to the total number of possible interactions generated from the in-silico model. This includes; Human-Human interactions, Human-nCoV interactions, and nCoV-nCoV interactions.

### 3.3. Validation through the State-of-the-Art Dataset 

Gordon et al. [105] proposed a host–pathogen interaction dataset physically connected with the human cell by cloning, tagging, and expressing 27 out of 29 proteins using affinity-purification mass spectrometry. Up to 14 open-reading frames can be encoded by a 30-kb genome (ORFs). In order to create the 16 non-structural proteins (NSP1-NSP16) that make up the replicase transcriptase complex, ORF1a and ORF1ab encode polyproteins. This produces a dataset of 332 high-confidence host–pathogen protein–protein interaction networks. However, while validating our computational model, we discovered that the protein sequences provided by Gordon et al. do not have any mapping with the corresponding UniProt id. In our situation, we have exclusively focused on the SARS-CoV-2 proteins published on UniProt. We have used a mathematical model to determine the binding affinities of a portion of the evaluated human proteins listed on UniProt. Because SARS-CoV-2 proteins could not be directly mapped into corresponding UniProt accession ids, direct comparison and validation concerning Gordon et al. were impossible. Thus, the nCoV proteins from Gordon et al. were mapped to the corresponding UniProt ids. As our research heavily depends on the underlying GO network of the host–pathogen protein interaction network, those proteins are selected with all three GO annotations. To validate our proposed method, all possible interactions are computed in our proposed computational environment, which gives 57,615 possible interactions, which are their respective fuzzy score from 27 bait and 332 prey. Among these interactions, 129 existing host–pathogen from high confidence dataset proposed by Gordon et al. whose scores are calculated. 

Apart from the high-confidence host–pathogen protein interaction network dataset, Gordon et al. also provided a host–pathogen interaction dataset that contains a human-nCoV protein interaction network without any threshold. This mainly contains scoring results of all bait and all prey proteins showing spectral counts of experimental samples. The dataset contains 22,153 interactions, including 27 bait and 2753 host proteins. Our proposed model generates an interaction network with the said protein, which generates all-vs-all interactions. Among those 22,153 interactions, there are 7866 existing host–pathogen interactions whose scores are calculated. Table 5 gives detailed information regarding the host–pathogen interaction for the high-confidence human–nCoV dataset and the generic human–nCoV dataset proposed by Gordon et al.

#### 3.3.1. Comparison with Gordon et al.

To validate our computational model, we compare our data set with that proposed by Gordon et al. [107]. To experiment with our proposed computational model, we construct a dataset of human and SARS-CoV-2/nCoV proteins retrieved from the UniProt protein repository, as discussed above. The computation results in fuzzy scoring of the protein pair (*viz.* human–human ppin, human–nCoV ppin, and nCoV–nCoV ppin). The edge-overlapping has shown the validation of our computational model between two datasets at different threshold values set on the fuzzy score. Edge overlapping signifies the common edges present in both datasets. For our experiment, we have kept the fuzzy score threshold ranging from 0.1–0.001. At first, we compare our network with the high-confidence human–nCoV network proposed by Gordon et al. The dataset contains 332 host proteins and 27 viral proteins. Table 6 compares two datasets at different threshold values and produces the intersected nodes and edges between the two datasets, along with the common host and viral proteins. 

The high-confidence dataset and the other dataset proposed by Gordon et al., which contains scoring results of all bait and all prey proteins showing spectral counts of experimental samples, are also being compared in the same manner discussed above with varying threshold values imposed on fuzzy interaction affinity score. The threshold ranges from 0.1–0.001. The dataset proposed by Gordon et al. contains 2753 host proteins and 27 viral proteins. Table 7 represents the comparison between the two datasets at different threshold values and produces the intersected nodes and intersected edges between the two datasets.

#### 3.3.2. Comparison with Dick et al.

Protein-protein Interaction Prediction Engine (PIPE) is a sequence-based PPI prediction approach that looks at sequence windows on each query protein proposed by Dick et al. [108]. The evidence for the putative PPI is strengthened if the two sequence windows have a lot in common with other pairs of proteins that have been found to interact. Normalization is used in a similarity-weighted (SW) scoring system to consider common sequences unrelated to PPIs. A PPI is anticipated, given enough supporting data [109,110,111]. For understudied species, the Protein-protein Interaction Prediction Engine (PIPE4) iteration has recently been modified [112].

Like PIPE, the SPRINT predictor gathers data from previously reported PPI interactions based on window similarity with the query protein pair to determine its prediction scores [113]. SPRINT uses a spaced seed method to compare the sequences of protein windows, where only certain places in the two windows must match, as determined by the bits of the spaced seeds. Additionally, because proteins are encoded with five bits per amino acid, it is possible to quickly compute protein window similarities and, consequently, forecast scores using very efficient (SIMD) bitwise operations [113].

Here, the two datasets produced by Dick et al. [108] are being compared, and an interaction affinity pair is being generated by using our proposed method. Table 8 shows the details of the comparison with both datasets. The table shows that PIPE4 contains 702 interactions, among which our proposed model identifies 575 interactions, and the score has been generated. On the other hand, the SPRINT dataset contains 510 interactions, among which 413 are identified by our proposed method. 

### 3.4. Vulnerable Host Protein

**Figure 2:**
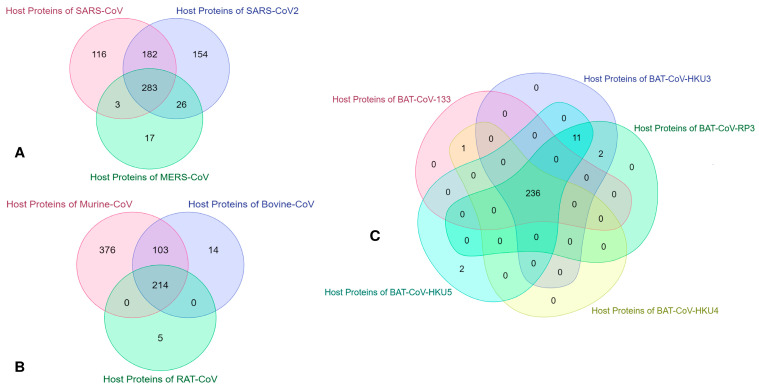
Venn diagram of the number of vulnerable host proteins obtained from host–pathogen interaction for all selected coronavirus organisms at 0.1 fuzzy threshold value. (**A**). The intersection of host protein identified from SARS-CoV-2, SARS-CoV, and MER-CoV. (**B**). Intersected host proteins from Murine-CoV, Bovine-CoV, and Rat Coronavirus. (**C**). Intersected host proteins of different viral organisms of Bat Coronavirus.

**Figure 3:**
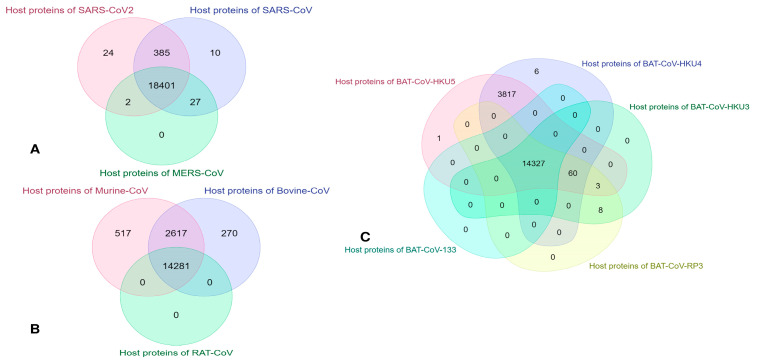
Venn diagram of the number of vulnerable host proteins obtained from host–pathogen interaction for all selected coronavirus organisms at 0.001 fuzzy threshold value. (**A**). Intersection of host protein identified from SARS-CoV-2, SARS-CoV, and MER-CoV. (**B**). The intersected host proteins from Murine-CoV, Bovine-CoV, and Rat Coronavirus. (**C**). Intersected host proteins from different viral organisms of Bat Coronavirus.

One of the main focuses of our research is to identify the common vulnerable host proteins at different threshold values. As discussed in Section 3.1, our computational model efficiently computes the interaction affinity and can generate a fuzzy score for any host–pathogen interaction pair for any organism from the corona family. We have experimented with the host–pathogen network for the entire corona family (with the selected organism, as mentioned in Section 2.2) and retrieved the network at different threshold values ranging from 0.1–0.001 at each threshold score, we segregate the network for each covid organism and construct their respective networks. Thus, for each threshold score, we obtained a separate host–pathogen network for each coronavirus organism. So, for each threshold score, some common host protein interacts with all the coronavirus organisms. As the value of the score decreases from a high threshold to a low threshold value, the number of common host proteins increases. These host proteins are the level one spreader nodes. These spreader nodes are identified by fuzzy thresholding, and these host proteins are vulnerable to the propagation or contamination of the diseases caused by the viral proteins. Table 9 represents the number of vulnerable host proteins at different fuzzy threshold scores. Figure 2 and Figure 3 represent the Venn diagram of the vulnerable host proteins at 0.1 and 0.001 threshold values, respectively. For simplicity and ease of the process, we divide the viral organism into three subsets. SARS-CoV-2, SARS-CoV and MERS-CoV forms one group, all the different organism from BAT-CoV (viz., *Bat coronavirus* HKU3, *Bat coronavirus* Rp3/2004, *Bat coronavirus* HKU5, *Bat coronavirus* HKU4, *Bat coronavirus* 133/2005) forms one group, and Murine-CoV, Bovine-CoV and Rat Coronavirus forms the third group. Then we identified the common host proteins from all three groups separately. Intersected host protein sets from all three groups are identified and again intersected. This results in the common vulnerable host proteins at the specified threshold value. For visualization, we only arbitrarily select a threshold value of 0.1 for constructing the Venn diagram, 0.1 threshold value gives 191 vulnerable host proteins interacting with all selected coronavirus organisms.

### 3.5. Identification of Potential Candidate FDA Drugs concerning Vulnerable Host-Proteins Using Human–Coronavirus Family Interaction Network Analysis

All level one human proteins of the coronavirus family are mapped with their matching medicines from DrugBank once the coronavirus family–human PIN has been created [114]. DrugBank is an online database that offers extensive information on medicines, drug-protein targets, and drug metabolism [115]. Most in-silico approaches used in drug design, drug docking, and drug interaction prediction use DrugBank as their most frequently used database because of its high-quality annotation. 

It has around 60% of FDA-approved medications and 10% of investigational drugs. It has been determined through adequate analysis that some spreader nodes in COVID19-human PPIN are the protein targets of possible COVID-19 FDA-listed medicines [116]: hydroxychloroquine [117], azithromycin [117], lopinavir [118], remdesivir [119,120], etc. Not only the list of drugs for COVID-19, but we have obtained a list of FDA-approved drugs from level 1 vulnerable host proteins for the entire coronavirus family by using Drug Consensus Score algorithm (DCS). The algorithm is defined as the number of times a drug occurs at a specific PPIN level. Each human protein is mapped with the appropriate related medicines in this level 1 PPIN.

The DCS, or frequency of each drug, is therefore calculated. Table 9 represents the top-5 FDA-approved drug at different fuzzy threshold values and the number of vulnerable host proteins at that corresponding threshold value, Drug ID, and corresponding DCS score for each drug. Fostamatinib is thought to be a promising medication for the target nCoV protein in the randomly created COVID-19 human PPI since it has the highest DCS in most cases.

## 4. Discussion

The number of vulnerable host proteins at different threshold values is represented in Table 10, and the list of the top five drugs, along with their drug-id based on the DCS score, are listed. This leads us to the analysis with the application of the lowest threshold values (i.e., 0.001), based on which the possible repurposed drugs are proposed.

Drug repurposing is a powerful strategy that gives new therapeutic alternatives by identifying other uses for already-approved medications, as vaccine and drug development can take years [121]. The traditional conservative drug development approach, which is restricted to “one drug, one target” paradigms, does not take into account or assess the off-target effects or the likelihood of numerous drug indications, even though some of them have since been confirmed to exist [122]. Upon the formation of the coronavirus–human PPIN, all level one Coronavirus human proteins are mapped with the appropriate medications via DrugBank [114]. DrugBank is an online database that provides detailed information on pharmaceuticals, drug-protein targets, and drug metabolism. DrugBank is the most often utilized database in practically all in silico approaches used in drug design, drug docking, and drug interaction prediction because of the high-quality annotation in the database. It includes 10% and 60% of FDA-approved and investigational medications [114]. It is observed that the above list of drugs at the threshold value 0.001, listed in Table 9, when compared to the remaining human protein-associated medications, fostamatinib has the highest frequency of occurrence in the entire PPIN and has a sizable overlap of target proteins in the human–coronavirus PPIN with highest Drug Consensus Score of 181. It was already discussed and proposed in [115] that Fostamatinib has the highest DCS score with reference to level one and level two human spreader proteins. Thus, our drug of concern shifted to the one with the next highest score, copper. Copper has an enormous effect in defeating COVID-19, which helps it to dominate with a high DCS score. The study proposed in [120] aims to investigate the effects of a highly specialized drug, “Hinokitiol Copper Chelate”, on enormous quantities of 2019-nCoV Spike Glycoprotein with a single receptor binding domain. This investigation offers a superior version of Hinokitiol Copper Chelate for in vitro testing against 2019-nCoV Main Protease. The authors suggest combining copper, NAC, colchicine, NO, and the experimental antivirals remdesivir or EIDD-2801 as a potential treatment for SARS-COV-2 [123]. In-silico docking study of copper complexes with SARS-CoV-2 viruses shows a steady binding with SARS-CoV-2 main protease (M^pro^) active-site region [124]. 

Zinc supplements also play a crucial role in combating different organisms of coronavirus. The essentiality of Zinc lies in the preservation of natural tissue barriers such as the respiratory epithelium, preventing pathogen entry for a balanced functioning of the human immune system. The deficiency of Zinc can probably lead to the infection and detrimental progression of COVID-19 [125]. The body’s tissue barriers, which contain cilia, mucus, anti-microbial peptides like lysozymes, and interferons, stop infectious organisms from entering. The primary mechanisms for SARS-CoV-2 entering cells are the cellular protease TMPRSS2 and the angiotensin-converting enzyme 2 (ACE2) [126]. People with COVID-19 are accompanied by ciliated epithelium destruction and ciliary dyskinesia, which limit mucociliary clearance [127]. The quantity and length of bronchial cilia increased after Zinc supplementation in Zinc-deficient rats [128].

In COVID-19, Zinc supplementation was hypothesized to reduce mortality. Supplementing with Zinc had no positive effects on how the illness progressed. The Zinc-supplemented group’s hospital stay was lengthier. There is no evidence to back up regular Zinc supplementation in COVID-19 [129]. The confounding variables impacting Zinc’s bioavailability may be avoided by administering Zinc intravenously, enabling Zinc to fulfill its medicinal potential. If effective, intravenous Zinc might be quickly incorporated into clinical practice due to benefits such as lack of toxicity, cheap cost, and accessibility of supply [130].

Promethazine, an antipsychotic agent showing clathrin-mediated endocytosis, is one most effective drugs for SARS-CoV and MERS-CoV, which has been repurposed for the treatment of COVID-19 as there is almost 89% genetic similarity with SARS-CoV-2 and SARS-CoV [131]. Two pills were offered as an intervention, one with Aspirin and Promethazine and the other with vitamins D3, C, and B3, together with Zinc and selenium supplements [132]. A randomized clinical trial has been conducted to recover mildly to moderate COVID-19 patients. 

Based on this validation, further research on the repurposed drug, docking study, and other symptomatic analyses will help to identify the potential drug for the entire coronavirus family. A clinical study on Promethazine and Fostamatinib [115,132] is also in progress. Even though the research is in its early stages, it in some way partially corroborates our findings.

## 5. Conclusions

Finding spreader nodes in any network of host–pathogen interactions is essential for predicting the course of a disease. However, not every protein in a network of interactions is highly capable of transmitting illness. In this work, we used the host–pathogen protein interaction network between humans and different coronavirus family organisms. Based on the available GO annotations of the proteins, a fuzzy interaction affinity score has been proposed for all the host–pathogen interactions. Our proposed model was validated with the *state-of-the-art* dataset. It has been noticed from this assessment that the chosen human spreader nodes, indicated by our suggested model, emerge as the possible protein targets for the different organisms of coronavirus medications authorized by the FDA, which highlights the significance of this proposed work. 

The basic hypothesis of the work is listed as follows: (1) Between SARS-CoV and SARS-CoV-2, there is a genetic overlap of around 89%, which also results in a substantial overlap in spreader proteins between human–SARS-COV and human–SARS-COV2 protein-interaction networks [79]. Moreover, we have considered the viral proteins of 11 different coronavirus organisms based on the available GO notations. (2) A fuzzy scoring approach for finding a protein’s interaction affinity with another protein helped build the host–pathogen network. (3) The proposed in-silico can effectively identify the host–pathogen protein–protein interaction network for identifying potential candidate FDA drugs concerning vulnerable host–proteins. 

Our proposed in-silico method for identifying host–pathogen protein interaction networks has been validated through different *state-of-the-art* datasets. According to recent research by Gordon et al., who focused on the sequence analysis of SARS-CoV-2 isolates, 332 high-confidence SARS-CoV-2–human protein–protein interactions have been discovered. Using affinity-purification mass spectrometry, they determined the human proteins that were physically linked to each of the 26 of the 29 SARS-CoV-2 proteins after they had been cloned, tagged, and produced in human cells [107]. While validating our work with Gordon et al., we discovered that the SARS-CoV-2 protein sequences employed by Gordon et al. do not exactly correspond to the accessible UniProt accession ids when comparing their foundational work with ours. In our situation, we exclusively focused on the SARS-CoV-2 proteins published on UniProt. We used a mathematical model to analyze the binding affinities of a subset of the human proteins available on UniProt. Because SARS-CoV-2 proteins could not be directly mapped into matching UniProt accession ids, direct comparison and validation concerning Gordon et al. were impossible. However, using the COVID-19 UniProtKB reference database, an attempt has been made to map the UniProt ids of Gordon et al. SARS-CoV-2 proteins [120]. 

In addition, our approach is not directly deal with the classification problem and does not require prior knowledge of positive and negative interaction. Further, several experiments show that Gordon et al. do not detect all the significant human–nCoV interactions [133,134]. For example, the essential protein for entry into the human host, ACE2 and TMPRSS2, are surprisingly not found in Gordon et al. However, in most of the covid related studies, Gordon et al. are considered one of the gold standards in human–nCoV interactions. When we quantitatively compared our findings with Gordon et al., we primarily focused on estimating TPR (higher is better) and FNR (lower is better) over node and edge overlaps between the two networks using multiple fuzzy thresholds. In this assessment, we observed that the optimal TPR (0.71) and FNR (0.29) are obtained around the fuzzy threshold 0.01 for node intersections while comparing with Gordon et al. Likewise, optimal TPR (0.86) and FNR (0.14) for edge intersection are observed at 0.001.

The target proteins of the possible FDA medications for the coronavirus family coincide with the spreader nodes of the hypothesized human–coronavirus protein interaction network, which may highlight one of the study’s major findings. Based on the DCS score applied on vulnerable host proteins identified at different threshold values, we have proposed a list of FDA-approved drugs such as Fostamatinib, Copper, Zinc Acetate, Zinc Chloride, etc. Our previous research has proposed Fostamatinib as a potential drug for COVID-19. This analysis demonstrates that these spreader nodes have biological importance in transmitting illness. Additionally, it spurs us to do medication repurposing research which focuses on the fact that apart from Fostamatinib, Promethazine can also be one of the potential drug candidates for coronavirus-related diseases under clinical trials. In a nutshell, the proposed methodology forms a complete PPIN for humans and different coronavirus organisms and adds much more relevant biological information about existing drugs against SARS-CoV-2 through a drug-repurposing study done with proper assessment and in-depth computational study. 

## Figures and Tables

**Table 1:** Detailed description of proteins and host–pathogen interaction for all organisms from the coronavirus family.

Organism	No. of Proteins	No. of Host–Pathogen Interaction
Severe acute respiratory syndrome coronavirus 2 (SARS-CoV-2)	14	205,140
Severe acute respiratory syndrome coronavirus (SARS-CoV)	15	233,411
*Bat coronavirus* HKU3	12	125,904
*Bat coronavirus* Rp3/2004	13	125,904
*Murine coronavirus*	40	425,162
Middle East respiratory syndrome-related coronavirus (MERS-CoV)	10	174,136
*Bovine coronavirus*	94	688,115
*Bat coronavirus* HKU5	10	117,090
Rat coronavirus	12	92,508
*Bat coronavirus* HKU4	10	117,090
*Bat coronavirus* 133/2005	10	98,494

**Table 2:** Detailed statistics of Human–nCoV protein interactions computed by our proposed model.

Intersection Type	Organism	Proteins	Interactions
All	Total Dataset	19,297	164,701,415
Host–Pathogen	Human–nCoV	19,297	206,516
Pathogen–-Pathogen	nCoV–nCoV	14	83
Host–Host	Human–Human	19,283	164,494,816

**Table 3:** Details of nCoV proteins collected from UniProt [106].

Entry	Entry Name	Gene Names	Protein Names
P0DTD1	R1AB_SARS2	rep 1a–1b	Replicase polyprotein 1ab, pp1ab (ORF1ab polyprotein)
P0DTC1	R1A_SARS2		Replicase polyprotein 1a, pp1a (ORF1a polyprotein)
P0DTC2	SPIKE_SARS2	S 2	Spike glycoprotein, S glycoprotein (E2) (Peplomer protein)
P0DTD8	NS7B_SARS2	7b	ORF7b protein, ORF7b (Accessory protein 7b)
P0DTC6	NS6_SARS2	6	ORF6 protein, ORF6 (Accessory protein 6)
P0DTC8	NS8_SARS2	8	ORF8 protein, ORF8 (Non-structural protein 8, ns8)
P0DTF1	ORF3B_SARS2		Putative ORF3b protein, ORF3b
P0DTC5	VME1_SARS2	M	Membrane protein, M (E1 glycoprotein
P0DTD3	ORF9C_SARS2	9c	Putative ORF9c protein, ORF9c
P0DTC3	AP3A_SARS2	3a	ORF3a protein, ORF3a
P0DTG0	ORF3D_SARS2		Putative ORF3d protein
P0DTG1	ORF3C_SARS2		ORF3c protein, ORF3c (ORF3h protein, ORF3h)
P0DTC7	NS7A_SARS2	7a	ORF7a protein, ORF7a
P0DTD2	ORF9B_SARS2	9b	ORF9b protein, ORF9b
P0DTC9	NCAP_SARS2	N	Nucleoprotein, N (Nucleocapsid protein, NC, Protein N)
P0DTC4	VEMP_SARS2	E 4	Envelope small membrane protein, E, sM protein

**Table 4:** Details of Human–nCov Interactions at different threshold values.

Interaction Type	Organism	Threshold	Nodes	Edges	Human	nCoV
Host–Pathogen	Human–nCoV	0.2	109	592	10	12
		0.15	245	1174	128	13
		0.1	886	2909	768	13
		0.09	1193	3586	1075	13
		0.08	1754	4619	1636	13
		0.05	7397	16,209	7278	13
		0.02	15,551	74,560	15,431	13
		0.001	18,936	166,382	18,816	14

**Table 5:** Overall statistics for interaction affinity score of High confidence Human–nCov dataset and all Human–nCov Dataset proposed by Gordon et al. computed by our proposed model.

Dataset	No. of Interactions	No. of Bait	No. of Prey	Total Interaction Score Computed
High Confidence Host–Pathogen PPI	332	27	332	57,615
All Host–Pathogen PPI	22,153	27	2,753	2,156,507

**Table 6:** Detailed validation of our model compared to High confidence human–nCoV proposed by Gordon et al.

HQ Data (Gordon et al.)	Our Dataset					
Number of Host	No. of Bait	Threshold	Number of Host	No. of Bait	No. of Intersected Nodes	No. of Intersected Edges
2753	27	0.1	17,875	13	88	149
2753	27	0.09	18,064	13	104	176
2753	27	0.08	18,218	13	128	214
2753	27	0.05	19,838	14	381	626
2753	27	0.02	19,123	14	1129	2513
2753	27	0.001	19,193	14	1817	6634

**Table 7:** Detailed validation of our model compared to all Human–nCov Datasets proposed by Gordon et al.

HQ Data (Gordon et al.)	Our Dataset					
Number of Host	No. of Bait	Threshold	Number of Host	No. of Bait	No. of Intersected Nodes	No. of Intersected Edges
332	27	0.1	768	13	8	5
332	27	0.09	1075	13	8	5
332	27	0.08	1636	13	8	5
332	27	0.05	7278	13	20	14
332	27	0.02	15,431	13	60	51
332	27	0.001	18,816	14	109	99

**Table 8:** Detailed validation of our model compared to all Human–nCov Datasets proposed by Dick et al.

Dataset (Dick et al.)	No. of Interactions	No. of Bait	No. of Prey	Total Interaction Score Computed
PIPE4	702	13	518	575
SPRINT	510	15	368	413

**Table 9:** Number of Vulnerable host proteins identified from the host–pathogen network for all selected coronavirus organisms at a different fuzzy threshold score.

Threshold	No. of Vulnerable Human Proteins
0.001	14,297
0.005	11,208
0.03	3889
0.05	526
0.07	351
0.1	191

**Table 10:** Top 5 target drugs with their respective DCS score at different threshold value.

Threshold	Vulnerable Human Proteins	Drug ID	DCS Score	Drug Name
0.001	14,297	DB12010	181	Fostamatinib
		DB09130	47	Copper
		DB14533	45	Zinc chloride
		DB14487	45	Zinc acetate
		DB01593	45	Zinc
0.005	11,208	DB12010	173	Fostamatinib
		DB01069	45	Promethazine
		DB01593	39	Zinc
		DB09130	39	Copper
		DB14487	39	Zinc acetate
0.03	3889	DB12010	25	Fostamatinib
		DB09130	6	Copper
		DB04464	5	N-Formylmethionine
		DB14487	5	Zinc acetate
		DB11638	5	Artenimol
0.05	526	DB12010	7	Fostamatinib
		DB12267	2	Brigatinib
		DB00041	2	Aldesleukin
		DB00074	2	Basiliximab
		DB09130	2	Copper
0.07	351	DB00041	2	Aldesleukin
		DB12010	2	Fostamatinib
		DB11638	2	Artenimol
		DB00004	2	Denileukin diftitox
		DB02240	1	Quinacrine mustard
0.1	191	DB12267	1	Brigatinib
		DB00111	1	Daclizumab
		DB11942	1	Selinexor
		DB08804	1	Nandrolone decanoate
		DB00047	1	Insulin glargine

## Data Availability

Data is available at the following GitHub link: https://github.com/SovanSaha/Assessment-of-GO-based-protein-interaction-affinities-in-the-3-large-scale-human-coronavirus-family.git (accessed on 1 February 2023) for free academic use.
